# Quality appraisal of radiomics-based studies on chondrosarcoma using METhodological RadiomICs Score (METRICS) and Radiomics Quality Score (RQS)

**DOI:** 10.1186/s13244-025-02016-3

**Published:** 2025-06-18

**Authors:** Salvatore Gitto, Renato Cuocolo, Michail E. Klontzas, Domenico Albano, Carmelo Messina, Luca Maria Sconfienza

**Affiliations:** 1 Dipartimento di Scienze Biomediche per la Salute, Università degli Studi di Milano, Milan, Italy; 2 IRCCS Istituto Ortopedico Galeazzi, Milan, Italy; 3 Department of Medicine, Surgery, and Dentistry, University of Salerno, Baronissi, Italy; 4 Artificial Intelligence and Translational Imaging (ATI) Lab, Department of Radiology, School of Medicine, University of Crete, Heraklion, Greece; 5 Department of Medical Imaging, University Hospital of Heraklion, Heraklion, Greece; 6 Division of Radiology, Department of Clinical Science, Intervention and Technology (CLINTEC), Karolinska Institutet, Stockholm, Sweden; 7 Dipartimento di Scienze Biomediche, Chirurgiche ed Odontoiatriche, Università Degli Studi di Milano, Milan, Italy; 8 UOC Radiodiagnostica, ASST Centro Specialistico Ortopedico Traumatologico Gaetano Pini-CTO, Milan, Italy

**Keywords:** Chondrosarcoma, Evidence-based radiology, Radiomics, Sarcoma, Texture analysis

## Abstract

**Objectives:**

To assess the methodological quality of radiomics-based studies on bone chondrosarcoma using METhodological RadiomICs Score (METRICS) and Radiomics Quality Score (RQS).

**Methods:**

A literature search was conducted on EMBASE and PubMed databases for research papers published up to July 2024 and focused on radiomics in bone chondrosarcoma, with no restrictions regarding the study aim. Three readers independently evaluated the study quality using METRICS and RQS. Baseline study characteristics were extracted. Inter-reader reliability was calculated using intraclass correlation coefficient (ICC).

**Results:**

Out of 68 identified papers, 18 were finally included in the analysis. Radiomics research was aimed at lesion classification (*n* = 15), outcome prediction (*n* = 2) or both (*n* = 1). Study design was retrospective in all papers. Most studies employed MRI (*n* = 12), CT (*n* = 3) or both (*n* = 1). METRICS and RQS adherence rates ranged between 37.3–94.8% and 2.8–44.4%, respectively. Excellent inter-reader reliability was found for both METRICS (ICC = 0.961) and RQS (ICC = 0.975). Among the limitations of the evaluated studies, the absence of prospective studies and deep learning-based analyses was highlighted, along with the limited adherence to radiomics guidelines, use of external testing datasets and open science data.

**Conclusions:**

METRICS and RQS are reproducible quality assessment tools, with the former showing higher adherence rates in studies on chondrosarcoma. METRICS is better suited for assessing papers with retrospective design, which is often chosen in musculoskeletal oncology due to the low prevalence of bone sarcomas. Employing quality scoring systems should be promoted in radiomics-based studies to improve methodological quality and facilitate clinical translation.

**Critical relevance statement:**

Employing reproducible quality scoring systems, especially METRICS (which shows higher adherence rates than RQS and is better suited for assessing retrospective investigations), is highly recommended to design radiomics-based studies on chondrosarcoma, improve methodological quality and facilitate clinical translation.

**Key Points:**

- The low scientific and reporting quality of radiomics studies on chondrosarcoma is the main reason preventing clinical translation.
- Quality appraisal using METRICS and RQS showed 37.3–94.8% and 2.8–44.4% adherence rates, respectively.
- Room for improvement was noted in study design, deep learning methods, external testing and open science.
- Employing reproducible quality scoring systems is recommended to design radiomics studies on bone chondrosarcoma and facilitate clinical translation.

**Graphical Abstract:**

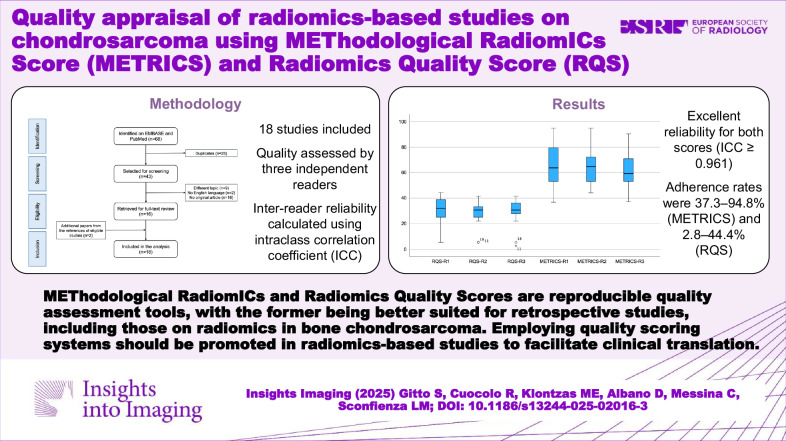

## Introduction

Chondrosarcoma is the most prevalent bone sarcoma in adults, accounting for 20–30% of primary malignant bone lesions [1]. Its incidence has markedly increased over the last three decades, particularly in the category of atypical cartilaginous tumor (ACT—formerly known as low-grade chondrosarcoma), due to an increase in incidental findings on MRI studies [2, 3]. According to the 2020 edition of the World Health Organization classification of bone tumors, ACT is categorized as an intermediate (locally aggressive) lesion located in long bones, which is low grade and shows relatively indolent clinical behavior with an unlikelihood to metastasize [4]. Cartilage lesions with the same histology as ACT, but located in the axial skeleton, are termed chondrosarcoma grade I and categorized in the malignant group, which also includes high-grade (II and higher) chondrosarcoma regardless of the axial or appendicular location [4]. Treatment strategies range from watchful waiting or intralesional curettage for ACT to wide resection for high-grade and axial grade I chondrosarcoma [5, 6]. However, accurate differentiation and grading of cartilage bone tumors are challenging for both radiologists and pathologists, particularly with intermediate lesions such as ACT in long bones, resulting in high interobserver variability even among experts [7–9].

Recent studies on chondrosarcoma have investigated the use of radiomics for both classification and prognostication purposes, such as grading or outcome prediction, with the number of publications exponentially growing in recent years [10]. Radiomics includes the extraction and analysis of quantitative features from medical images, known as radiomic features, which can be combined with machine learning models to predict the diagnosis or outcome of interest [11–13]. However, although radiomics holds great potential to augment clinical decision-making in cartilaginous bone tumors, its translation into clinical practice remains a challenge [14]. The low scientific and reporting quality of radiomics-based studies on chondrosarcoma is regarded as the main reason preventing clinical translation [10]. To address this issue, several guidelines, checklists and scoring systems have been proposed [15–18]. Particularly, Radiomics Quality Score (RQS) was proposed by Lambin et al in 2017 and has progressively become the most commonly employed scoring system to assess methodology and reporting quality of radiomics pipelines [17]. More recently, the European Society of Medical Imaging Informatics proposed the METhodological RadiomICs Score (METRICS) as an easy-to-use quality assessment tool to evaluate and improve methodology in radiomics-based studies [16].

The objectives of this investigation are to systematically review radiomics-based studies on bone chondrosarcoma and assess their methodological quality using METRICS and RQS. The ultimate goals are to promote robust radiomics pipelines and bridge the gap between radiomics research and real-life application, thus potentially helping clinicians to face the increasing detection rate of cartilaginous bone tumors in everyday practice.

## Methods

### Search strategy

No Ethical Committee approval was needed for this study, which was based on a systematic review of the literature and did not include any new patients’ data. This research was conducted according to the preferred reporting items for systematic reviews and meta-analyses (PRISMA) checklist [19]. Literature search and study selection were performed independently by three musculoskeletal radiologists with 3 to 7 years of experience in bone tumors and radiomics (S.G., C.M., D.A.). An electronic literature search was conducted on EMBASE (Elsevier) and PubMed (MEDLINE, US National Library of Medicine and National Institutes of Health) databases for studies on radiomics in bone chondrosarcoma, which were published up to July 31, 2024. A controlled vocabulary was adopted using medical subject headings in PubMed and the thesaurus in EMBASE. The exact search query was: (‘radiomics’/exp OR radiomics) AND (‘chondrosarcoma’/exp OR chondrosarcoma). The full text and supplementary material of eligible papers were retrieved for further review. Additionally, the references of eligible studies were checked for additional papers to include. Inclusion criteria were: (1) original research papers published in peer-reviewed journals; (2) research focused on radiomics applied to cartilaginous tumors of the bone, with no restrictions regarding the aim of the study; (3) statement that local ethics committee approval was obtained, or ethical standards were followed. Duplicates, studies on different topics and papers in languages other than English were excluded.

### Data extraction and scoring

Data extraction and scoring were performed by three readers with 7 to 10 years of experience in radiomics and artificial intelligence (R1 = S.G., R2 = R.C., R3 = M.E.K.). An introductory session was conducted to analyze and discuss the items of METRICS and RQS. Therefore, each of the three researchers was asked to read and independently evaluate all papers using both scoring tools, including supplementary material if available. METRICS consists of 30 items grouped into 9 categories [16]. It was calculated using the web application METRICS Tool v1.0 (https://metricsscore.github.io/metrics/METRICS.html), which generated a separate scoring sheet for each evaluated paper. RQS consists of 16 items, each with a corresponding number of points for a total of 36 [17], which were assigned and recorded in a scoring sheet. Both METRICS and RQS final scores were expressed as percentages. Additionally, baseline characteristics of all evaluated studies were extracted and summarized by the same readers, including the first author’s name, year of publication, journal, aim of the study, study design, reference standard, imaging modality and database size.

### Statistical analysis

Categorical variables were expressed as absolute values and percentages. Quantitative variables were expressed as median and interquartile (1st–3rd) range (IQR). Inter-reader reliability was calculated using intraclass correlation coefficient (ICC) with a two-way random effect, single rater, absolute agreement model. ICC values were interpreted as follows: ICC < 0.50 indicated poor reliability, 0.50 ≤ ICC < 0.75 indicated moderate reliability, 0.75 ≤ ICC < 0.90 indicated good reliability, and ICC > 0.90 indicated excellent reliability [20]. A two one-sided *t*-test (TOST) procedure was employed to compare equivalence and/or differences in the distribution of RQS and METRICS final scores. TOST was performed with an alpha level of 0.05, assuming equivalence bounds to be between −5% and +5% of the total score. Statistical analysis was performed using IBM SPSS Statistics (version 29.0) and R (version 4.3.2). A *p*-value < 0.05 was considered statistically significant.

## Results

### Literature search

**Fig. 1:**
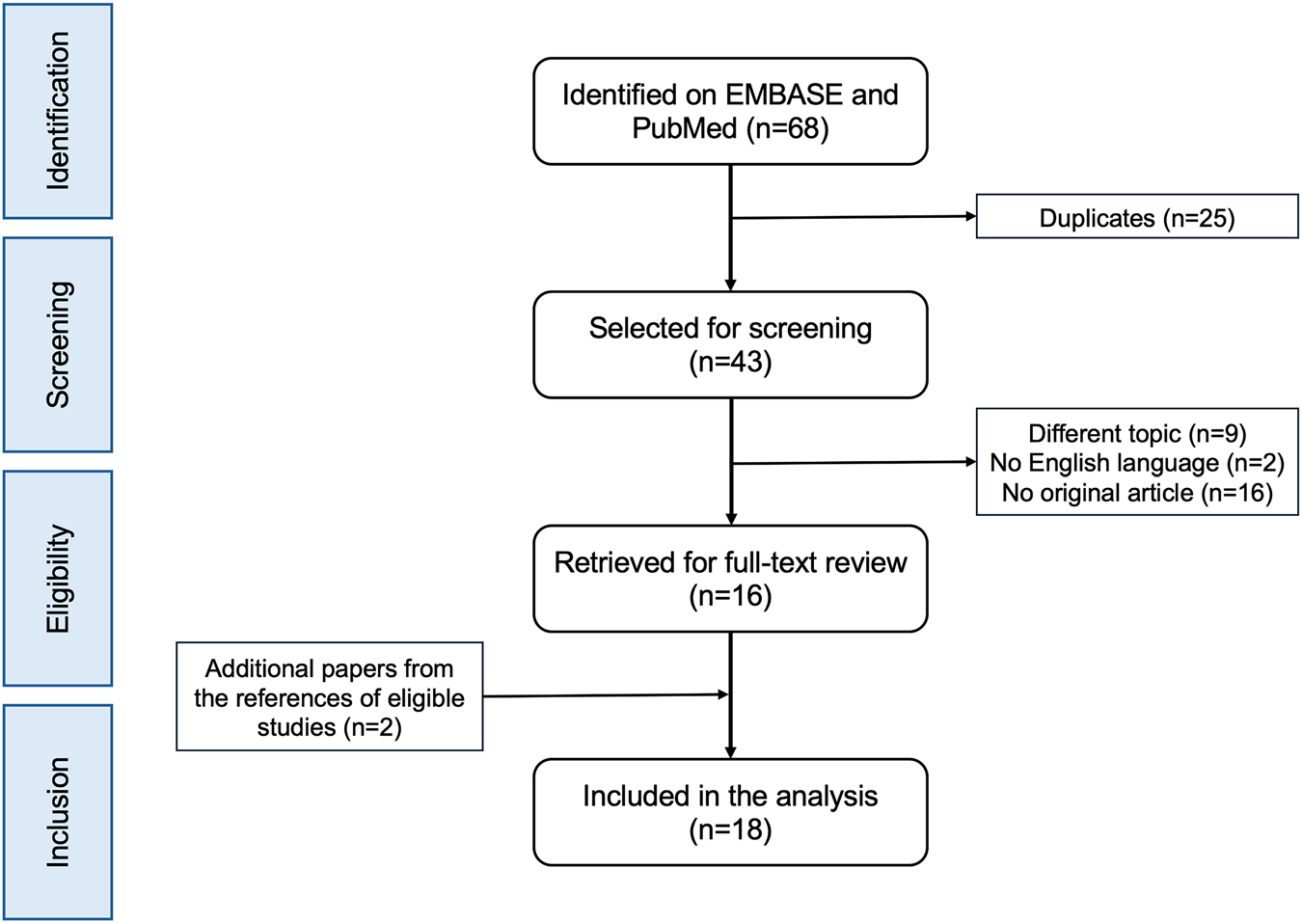
PRISMA (Preferred Reporting Items for Systematic reviews and Meta-Analyses) flowchart of systematic identification, screening, eligibility and inclusion information from retrieved studies

Sixty-eight papers were initially identified. Of these, 25 were duplicates. After removing non-original research papers (*n* = 16), studies on different topics (*n* = 9) or in languages other than English (*n* = 2) and identifying additional studies among the references of the eligible publications (*n* = 2), 18 papers were finally included in our analysis. A flowchart showing the literature search process is shown in Fig. 1.

### Baseline study characteristics

The baseline characteristics of the included studies are summarized in Table 1. Nine (50%) out of 18 included studies were published between 2023 and 2024, 5 (28%) between 2021 and 2022, and 4 (22%) between 2019 and 2020. Both classification (diagnosis-related) and prognostication (outcome-related) studies were included. Classification tasks included benign vs malignant discrimination or grading of cartilaginous bone tumors (*n* = 13) and chondrosarcoma differentiation from other lesions, such as skull and sacral chordomas (*n* = 3). Prognostication tasks included prediction of relapse (*n* = 1) and survival (*n* = 2). Of note, one study aimed at predicting both survival and grade [21], as reported in Table 1. The design was retrospective in all studies. In classification studies, histology was the reference standard in all cases except 2 studies where benign lesions were diagnosed based on stable imaging features over time [22, 23] and another study reporting no reference standard [24]. In prognostication studies, survival was assessed based on clinical and radiological follow-up, and relapse was assessed based on histology or imaging findings. The most investigated imaging modalities were MRI in 13 (72%) and CT in 4 (22%) studies, respectively, with one study including both modalities in the radiomics workflow [25]. X-rays and SPECT were investigated in 1 study each. The median (IQR) database size was 103 (84–143) patients. None of the included studies employed public data.

**Table 1:** Baseline characteristics of the radiomics-based studies on bone chondrosarcoma

Authors	Year	Journal	Aim	Design	Reference standard	Modality	Dataset (*n*)
Amini B et al [35]	2023	JCO Precis Oncol	Differentiation from chordoma	Retrospective	Histology	MRI	82
Cilengir AH et al [22]	2023	Skelet Radiol	Benign vs malignant/grading	Retrospective	Histology Imaging follow-up (benign)	MRI	47
Deng XY et al [25]	2021	Front Oncol	Benign vs malignant/grading	Retrospective	Histology	CT MRI	91
Erdem F et al [36]	2023	J Clin Ultrasound	Benign vs malignant/grading	Retrospective	Histology	MRI	88
Fritz B et al [23]	2018	Invest Radiol	Benign vs malignant/grading	Retrospective	Histology Imaging follow-up (benign)	MRI	116
Gitto S et al [37]	2020	Eur J Radiol	Benign vs malignant/grading	Retrospective	Histology	MRI	58
Gitto S et al [38]	2021	EBioMedicine	Benign vs malignant/grading	Retrospective	Histology	CT	120
Gitto S et al [39]	2022	EBioMedicine	Benign vs malignant/grading	Retrospective	Histology	MRI	158
Gitto S et al [40]	2024	EBioMedicine	Benign vs malignant/grading	Retrospective	Histology	X-rays	150
Li L et al [41]	2019	Eur J Radiol	Differentiation from chordoma	Retrospective	Histology	MRI	210
Li Q et al [42]	2024	Insights Imaging	Survival	Retrospective	Clinical/imaging follow-up	CT	214
Li X et al [43]	2023	Front Oncol	Benign vs malignant/grading	Retrospective	Histology	MRI	102
Li X et al [21]	2024	Cancer Imaging	Benign vs malignant/grading Survival	Retrospective	Histology (grading) Clinical/imaging follow-up (survival)	CT	196
Li X et al [44]	2024	BMC Med Imaging	Benign vs malignant/grading	Retrospective	Histology	MRI	114
Pan J et al [45]	2021	J Magn Reson Imaging	Benign vs malignant/grading	Retrospective	Histology	MRI	103
Yamazawa E et al [46]	2022	Cancers	Differentiation from chordoma	Retrospective	Histology	MRI	57
Yin P et al [47]	2020	J Magn Res Imaging	Relapse	Retrospective	Histology Imaging	MRI	103
Yoon H et al [24]	2023	Tomography	Benign vs malignant/grading	Retrospective	n/a	SPECT-CT	49

### Radiomics scoring

**Fig. 2:**
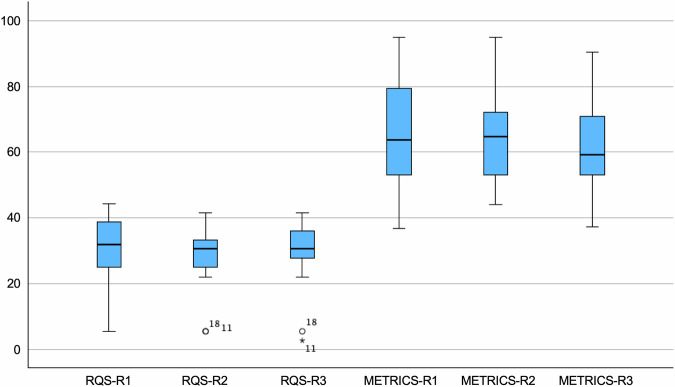
Boxplot comparison of METRICS and RQS final scores, presented as percentages, for all three readers

**Fig. 3:**
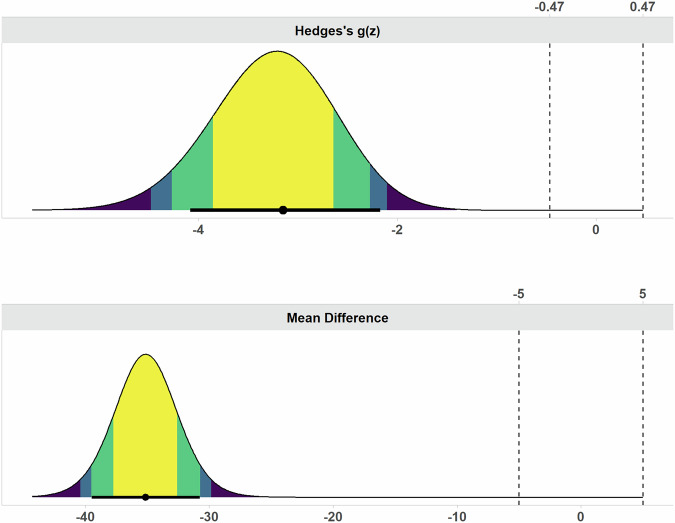
Effect size (Hedges’s g) and mean difference plots depicting the results of the equivalence test between METRICS and RQS final scores

METRICS and RQS final scores of each evaluated paper are reported for all readers in Table 2. In detail, METRICS and RQS final scores ranged between 37.3–94.8% and 2.8–44.4%, respectively. ICC values indicated excellent inter-reader reliability for both METRICS and RQS, as shown in Fig. 2. In detail, ICC values were 0.961 (95% confidence interval: 0.916–0.984) for METRICS and 0.975 (95% confidence interval: 0.944–0.990) for RQS, respectively. Tables 3 and 4 summarize the METRICS and RQS results scored overall in the included papers and grouped by item, respectively. The TOST procedure resulted in no equivalence between METRICS and RQS final scores, within −5% to +5% bounds (*p* > 0.99). Figure 3 shows the mean difference and effect size (i.e., Hedges’s g) obtained via TOST.

**Table 2:** METRICS and RQS final scores reported for all three readers

	METRICS scoring			RQS scoring		
	R1	R2	R3	R1	R2	R3
Amini B et al [35]	51.4	47.1	47.1	27.8	25	30.6
Cilengir HA et al [22]	36.8	54.0	54.0	25	22.2	22.2
Deng XY et al [25]	55.1	55.6	55.6	25	25	30.6
Erdem F et al [36]	39.3	48.9	54.2	5.6	5.6	2.8
Fritz B et al [23]	47.7	44.2	37.3	8.3	5.6	5.6
Gitto S et al [37]	60.7	60.7	61.2	30.6	30.6	27.8
Gitto S et al [38]	87.2	84.0	84.0	41.7	41.7	41.7
Gitto S et al [39]	87.2	79.5	85.6	44.4	38.9	38.9
Gitto S et al [40]	94.8	94.8	90.4	38.9	38.9	38.9
Li L et al [41]	58.6	44.4	41.9	25	25	27.8
Li Q et al [42]	74.9	70.9	70.9	33.3	33.3	27.8
Li X et al [43]	68.7	68.7	57.5	36.1	33.3	41.7
Li X et al [21]	79.4	72.2	69.1	33.3	33.3	36.1
Li X et al [44]	83.2	85.6	82.5	38.9	30.6	33.3
Pan J et al [45]	60.0	64.4	69.7	38.9	30.6	33.3
Yamazawa E et al [46]	67.0	67.0	52.2	33.3	27.8	25
Yin P et al [47]	71.5	65.3	65.3	30.6	30.6	36.1
Yoon H et al [24]	53.2	53.2	53.2	27.8	27.8	27.8

**Table 3:** Overall METRICS scoring results for each item and condition in radiomics-based studies on bone chondrosarcoma, as rated by R1 (S.G.) [16]

METRICS items, conditions and definitions		Yes (*n*)	N/A (*n*)	No (*n*)	Yes (%)
Study design					
Item #1	Adherence to radiomics and/or machine learning-specific checklists or guidelines	2	0	16	11.1
Item #2	Eligibility criteria that describe a representative study population	17	0	1	94.4
Item #3	High-quality reference standard with a clear definition	13	0	5	72.2
Imaging data					
Item #4	Multi-center	6	0	12	33.3
Item #5	Clinical translatability of the imaging data source for radiomics analysis	18	0	0	100
Item #6	Imaging protocol with acquisition parameters	14	0	4	77.8
Item #7	The interval between imaging used and reference standard	12	0	6	66.7
Segmentation					
Condition #1	Does the study include segmentation?	18	0	0	100
Condition #2	Does the study include fully automated segmentation?	0	0	18	0
Item #8	Transparent description of segmentation methodology	18	0	0	100
Item #9	Formal evaluation of fully automated segmentation	0	18	0	0
Item #10	Test set segmentation masks produced by a single reader or automated tool	7	0	11	38.9
Image processing and feature extraction					
Condition #3	Does the study include hand-crafted feature extraction?	18	0	0	100
Item #11	Appropriate use of image preprocessing techniques with transparent description	12	0	6	66.7
Item #12	Use of standardized feature extraction software	11	0	7	61.1
Item #13	Transparent reporting of feature extraction parameters, otherwise providing a default configuration statement	15	0	3	83.3
Feature processing					
Condition #4	Does the study include tabular data?	18	0	0	100
Condition #5	Does the study include end-to-end deep learning?	0	0	18	0
Item #14	Removal of non-robust features	10	0	8	55.6
Item #15	Removal of redundant features	17	0	1	94.4
Item #16	Appropriateness of dimensionality compared to data size	15	0	3	83.3
Item #17	Robustness assessment of end-to-end deep learning pipelines	0	18	0	0
Preparation for modeling					
Item #18	Proper data partitioning process	15	0	3	83.3
Item #19	Handling of confounding factors	9	0	9	50
Metrics and comparison					
Item #20	Use of appropriate performance evaluation metrics for task	18	0	0	100
Item #21	Consideration of uncertainty	11	0	7	61.1
Item #22	Calibration assessment	7	0	11	38.9
Item #23	Use of uni-parametric imaging or proof of its inferiority	14	0	4	77.8
Item #24	Comparison with a non-radiomic approach or proof of added clinical value	12	0	6	66.7
Item #25	Comparison with simple or classical statistical models	0	0	18	0
Testing					
Item #26	Internal testing	12	0	6	66.7
Item #27	External testing	5	0	13	27.8
Open science					
Item #28	Data availability	1	0	17	5.6
Item #29	Code availability	3	0	15	16.7
Item #30	Model availability	8	0	10	44.4

**Table 4:** Overall RQS scoring results for each item in radiomics-based studies on bone chondrosarcoma, as rated by R1 (S.G.) [17]

RQS items and definitions	Points scored (*n*)	Max points achievable (*n*)	Points scored (%)
Item #1—Image protocol quality (from 0 to +2 per study)	14	36	38.9
Item #2—Multiple segmentations (from 0 to +1 per study)	12	18	66.7
Item #3—Phantom study on all scanners (from 0 to +1 per study)	0	18	0
Item #4—Imaging at multiple time points (from 0 to +1 per study)	0	18	0
Item #5—Feature reduction or adjustment for multiple testing (from −3 to +3 per study)	54	54	100
Item #6—Multivariable analysis with non-radiomics features (from 0 to +1 per study)	8	18	44.4
Item #7—Detect and discuss biological correlates (from 0 to +1 per study)	18	18	100
Item #8—Cut-off analyses (from 0 to +1 per study)	2	18	11.1
Item #9—Discrimination statistics (from 0 to +2 per study)	28	36	77.8
Item #10—Calibration statistics (from 0 to +2 per study)	7	36	19.4
Item #11—Prospective study registered in a trial database (from 0 to +7 per study)	0	126	0
Item #12—Validation (from −5 to +5 per study)	28	90	31.1
Item #13—Comparison to ‘gold standard’ (from 0 to +2 per study)	12	36	33.3
Item #14—Potential clinical utility (from 0 to +2 per study)	4	36	11.1
Item #15—Cost-effectiveness analysis (from 0 to +1 per study)	0	18	0
Item #16—Open science and data (from 0 to +4 per study)	9	72	12.5

## Discussion

In the present study, we evaluated the methodological quality of radiomics-based studies on chondrosarcoma using METRICS and RQS. Most analyzed studies addressed clinical questions related to the diagnosis and grading of cartilaginous bone tumors, while a few papers focused on outcome prediction. Overall, METRICS and RQS highlighted some drawbacks in current radiomics pipelines, which should be addressed in future investigations to improve methodology and promote translation into clinical practice. Additionally, both scores had excellent inter-reader reproducibility, underscoring their reliability as quality assessment tools in radiomics research.

METRICS adherence rates were higher than RQS in the evaluated papers, ranging between 37.3% and 94.8%. Some weaknesses were identified, including the lack of fully automated segmentation processes, deep learning pipelines and comparison with statistical methods and the limited adherence to radiomics or machine learning guidelines, external testing and availability of open science data. In musculoskeletal oncology, fully automated segmentation and deep learning pipelines were investigated less than manual image segmentation and conventional machine learning approaches, respectively [26]. However, promising results were achieved in preliminary studies on osteosarcoma and soft-tissue sarcomas [27–29], thus highlighting the need for further efforts in this direction. A comparison between radiomics models and statistical methods is also important to establish whether the former offer significant advantages over traditional statistical approaches. Additionally, although an improvement was recently reported [30], the number of studies including an external dataset for independent testing is still suboptimal (27.8% according to our analysis), therefore limiting the generalizability of radiomics models. Finally, the limited availability of open science data, codes and models remains an unsolved issue, which may, however, be addressed by initiatives aimed at establishing public databases for radiomics-based studies [31].

Low RQS adherence rates were found in radiomics-based studies on bone chondrosarcoma, ranging between 2.8% and 44.4%. The absence of studies with prospective design, phantom studies, imaging evaluation at multiple time points and cost-effectiveness analyses was reported in all evaluated papers. Additionally, the insufficiency of cut-off analyses, calibration statistics, clinical utility analyses and open science data was repeatedly addressed. These findings are in line with a previous systematic review on radiomics in bone chondrosarcoma, which highlighted similar issues [10].

Although the two scores cannot be compared directly, RQS adherence rates were lower than METRICS in the analyzed studies, which is in keeping with previous reports on radiomics quality assessment in endometrial [32] and prostate [33] cancers. This is at least partially attributable to the different item weight distribution in the two scores, which is focused on a few items in RQS. Indeed, the relative weight of some RQS items (e.g., +7 points for prospective study design) may penalize preliminary investigations and studies focusing on rare lesions such as skeletal sarcomas, where a retrospective design is often chosen to include enough patients in data analysis. Conversely, METRICS includes a step-by-step quality assessment process with conditional questions, shows a more balanced item weight distribution than RQS and allows handling various study designs, thus resulting in different final score distributions between the two scores.

Some limitations of the present study should be addressed. First, the number of research papers on radiomics in bone chondrosarcoma has been limited to date, resulting in a relatively small number of publications included in our analysis. Additionally, most of the analyzed studies were published before the introduction of METRICS, which may serve as a guide in future research papers and therefore improve overall methodological quality. Finally, the three readers involved in paper scoring were all experienced in radiomics, and two of them (R.C. and M.E.K.) were also METRICS developers. As the application of radiomics scoring tools is challenging for inexperienced readers [34], interobserver reliability could be lower if papers were evaluated by less expert raters.

In conclusion, both METRICS and RQS are reproducible quality assessment tools in radiomics-based studies on bone chondrosarcoma, with variable adherence rates found in current studies. Particularly, quality appraisal based on METRICS resulted in higher adherence rates than RQS, as the former includes a more balanced item weight distribution and is better suited for assessing papers with retrospective design, which is often chosen when dealing with rare lesions such as skeletal sarcomas. Employing quality scoring systems, especially METRICS, should be promoted as a step-by-step guide to design future radiomics-based studies on cartilaginous bone tumors, improve methodological quality and facilitate clinical translation.

## Data Availability

Data generated or analyzed during this study are presented with this manuscript.

## Abbreviations

ACT: Atypical cartilaginous tumor
ICC: Intraclass correlation coefficient
IQR: Interquartile range
METRICS: METhodological RadiomICs Score
PRISMA: Preferred reporting items for systematic reviews and meta-analyses
RQS: Radiomics quality score
TOST: Two one-sided *t*-test
